# Diagnosing Severe Low-Gradient vs Moderate Aortic Stenosis with Artificial Intelligence Based on Echocardiography Images

**DOI:** 10.1007/s10278-025-01497-4

**Published:** 2025-04-21

**Authors:** Michał Wrzosek, Mikolaj Buchwald, Patryk Czernik, Szymon Kupinski, Karina Zatorska, Anna Jasińska, Dariusz Zakrzewski, Juliusz Pukacki, Cezary Mazurek, Robert Pękal, Tomasz Hryniewiecki

**Affiliations:** 1 Department of Valvular Heart Disease, National Institute of Cardiology, Warsaw, Poland; 2 Poznan Supercomputing and Networking Center, Polish Academy of Sciences, Poznan, Poland

**Keywords:** Aortic stenosis, Low-gradient aortic stenosis, Artificial intelligence, Echocardiography, Clinical decision support system

## Abstract

Diagnosis of aortic valve stenosis (AS) is performed manually by a physician experienced in echocardiography imaging. A specific subtype of AS, a severe low-gradient AS, is the most challenging one in terms of differentiating it from the moderate AS. In this study, an artificial intelligence (AI)-based model was used to diagnose the severe low-gradient AS in a fully automatic manner. Data from 158 consecutive patients undergoing echocardiography examination to assess AS severity were used. The obtained performance of our fully automatic approach was AUC = 0.719, 95% confidence interval, 0.640–0.798. It is an important step towards a comprehensive and automatic, image-only-based clinical decision support system for determining the presence of AS and its severity, especially in AS subtypes, such as low-gradient AS.

**Supplementary Information:**

The online version contains supplementary material available at 10.1007/s10278-025-01497-4.

## Introduction

Aortic valve stenosis (AS) is the most common valvular heart disease in Europe and North America, affecting up to 10% of the population by the eighth decade [1] and 9 million people worldwide [2]. Depending on which symptoms are present, AS becomes fatal in 2 to 5 years [3]. The only effective treatment is to perform the invasive intervention in severe AS [4]. Typically, the severe AS is correlated with mean aortic gradient (mAG) > 40 mmHg; however, in the case of the severe low-gradient (LG) AS, mAG is below 40 mmHg, suggesting moderate stenosis. This makes differentiation between low-gradient severe and moderate AS substantially more difficult by conventional diagnostic means. AS is characterized by the progressing narrowing of the aortic valve area (AVA). There is an acceleration of the narrowing as the disease advances [5]. To compensate for the stenosis, the left ventricle becomes hypertrophic. This process is strongly correlated to AS, but it is not specific to this disease entity. Although there were already attempts to improve AS diagnosis with AI algorithms, such studies were restricted primarily to differentiating between severe AS cases and non-diseased individuals [6–8].

Previously, it has been shown that biomarkers developed based on analysis of simple parasternal long-axis (PLAX) videos allow stratification of the risk of AS occurrence and progression [9]. Duffy et al. developed a model that performs frame-level semantic segmentation of the left ventricular wall thickness from parasternal long-axis echocardiogram videos that allows quantifying ventricular wall thickness [10]. EchoNet-Dynamic model, devised by the same group, performs semantic segmentation at the frame level throughout the heart cycle, and then, based on spatial and temporal dimensions, the model predicts ejection fraction [11, 12]. Holste et al. worked on an approach for detecting severe AS based on PLAX view and deep RES-net architecture [13]. However, all AS cases with low-flow and low-gradient were excluded from the training process. Thus, the classifier performance for severe low-gradient cases could not be assessed [13].

In virtually all previous studies using AI for AS diagnosis support, in addition to image-derived parameters, also non-imaging parameters such as clinical information or patient demographics information were used. Also, the goals of using AI applications for echocardiographic data, in the context of AS, were predominantly restricted to just diagnosing AS at all, not differentiating between particular degrees of its severity. Finally, current clinical guidelines suggest using multiple different medical procedures and examinations to evaluate LG AS severity and patient eligibility for intervention [14, 15]. In our work, we aimed to create an AI model based solely on imaging features derived from a single procedure (transthoracic echocardiography, TTE) that would reliably differentiate between the two very similar AS subtypes, namely, severe low-gradient AS and moderate AS.

## Materials and Methods

### Study Population

In the dataset used in this study, there were 174 consecutive patients with AS (97 male, 77 female) from National Institute of Cardiology in Warsaw. Of these, 91 patients were diagnosed with moderate AS, and 83 with severe low-gradient AS (Table 1). Patients with atrial fibrillation (AF) present during echocardiographic examination were included in the study, as the measurements were averaged over 5 heartbeats, in which case the AF is not a confounding factor. The severe AS cases, depending on characteristics of the severity, were confirmed with one or more of the following additional diagnostic procedures: dobutamine stress echocardiography (DSE), computed tomography (CT), or transesophageal echocardiography (TEE), and were deemed eligible for intervention by the Heart Team. The Institutional Bioethical Committee of National Institute of Cardiology in Warsaw reviewed and approved the study procedures, decision no. 1967.

**Table 1:** Patient characteristics for low-gradient severe and moderate aortic stenosis

Variable	Moderate AS (*n* = 91)	Severe low-gradient AS (*n* = 83)	*p* value
Age, median (IQR)	71 (62–78)	74 (68–81)	< 0.05
Female, *n* (%)	33 (36%)	44 (53%)	< 0.05
Heart failure (LVEF < 50% *n* (%))	9 (10%)	29 (35%)	< 0.001
Hypertension, *n* (%)	70 (77%)	56 (67%)	0.2
Renal disease, *n* (%)	18 (20%)	24 (29%)	0.2
Diabetes mellitus, *n* (%)	21 (23%)	30 (36%)	0.059
Atrial fibrillation, *n* (%)	11 (12%)	20 (24%)	< 0.05
EF, median (IQR)	65 (60–67)	58 (35–65)	< 0.001
mAG, median (IQR)	23 (15.5–28.5)	30 (24.5–33.5)	< 0.001
IVS, median (IQR)	12 (11–14)	13 (11–14)	0.3
LVID, median (IQR)	47 (43–52)	47 (43–54)	0.4
SVI, median (IQR)	51 (44–56)	38 (28–45)	< 0.001
DVI, median (IQR)	0.32 (0.27–0.36)	0.22 (0.18–0.26)	< 0.001

*AS* aortic stenosis, *IQR* interquartile range, *LVEF* left ventricular ejection fraction, *EF* ejection fraction, *mAG* mean aortic gradient, *IVS* interventricular septum thickness in diastole, *LVID* left ventricular internal dimension in diastole, *SVI* stroke volume index, *DVI* Doppler velocity index

### Imaging Parameters for AI/ML

Our model incorporated in a multivariate manner only ventricular and valvular parameters, and no additional echocardiographic variables characterizing severe AS were used (such as AVA, mAG, or peak aortic jet velocity). We used 5 parameters describing the left ventricle: (1) a diastolic left ventricular internal diameter (LVID), (2) thickness of interventricular septum (IVS), (3) thickness of left ventricular posterior wall (LVPW), (4) left ventricular ejection fraction (LVEF), and (5) the stroke volume index (SVI). Additionally, one valvular parameter was used, namely, the Doppler velocity index (DVI). Three of the ventricular parameters, LVID, IVS, and LVPW, were obtained automatically with the previously published EchoNet-LVH model [10], utilizing the PLAX projection at full diastole (a single frame). Another left ventricle characteristic, LVEF, was also calculated automatically, but with a different model—EchoNet-Dynamic, and this time the apical four chamber (A4C) view was used (multiple frames) [12]. The final, fifth ventricular parameter, SVI, as well as the valvular parameter, DVI, were calculated manually by clinical experts based on tracing of the velocity–time curves (i.e., these parameters, which were used later only in the semi-automatic model/approach, were calculated manually by physicians). All parameters used in the further described analyses are summarized in Table 2.

**Table 2:** Parameters used in the study

No	Parameter name	Derived	Describing/characterizing
1	Left ventricular internal diameter (LVID)	Automatically	Left ventricle
2	Thickness of interventricular septum (IVS)	Automatically	Left ventricle
3	Thickness of left ventricular posterior wall (LVPW)	Automatically	Left ventricle
4	Stroke volume index (SVI)	Manually	Left ventricle
5	Doppler velocity index (DVI)	Manually	Aortic valve
6	Left ventricular ejection fraction (LVEF)	Automatically	Left ventricle

### Data Preprocessing

Outliers were removed for LVPW and IVS parameters using the two-sigma method, i.e., the values below and above two standard deviations in reference to the mean were removed.

### Selecting the Frame for Parameter Calculation

As EchoNet-LVH provides parameters for each analyzed frame, a data aggregation step was necessary (e.g., averaging or frame selection). According to the clinical guidelines, the LVID parameter should be chosen for diastole for a good-quality recording [16]. Due to the limited number of frames/patients being qualified when using these crude requirements and EchoNet’s information on phase, an additional algorithm was used for selecting the appropriate frame to obtain the parameters from. Namely, for each patient (i.e., echocardiography recording), a frame with the maximum value of the LVID parameter was selected, and for which additionally LVPW and IVS parameters were also available/calculated.

### Model Development

**Fig. 1:**
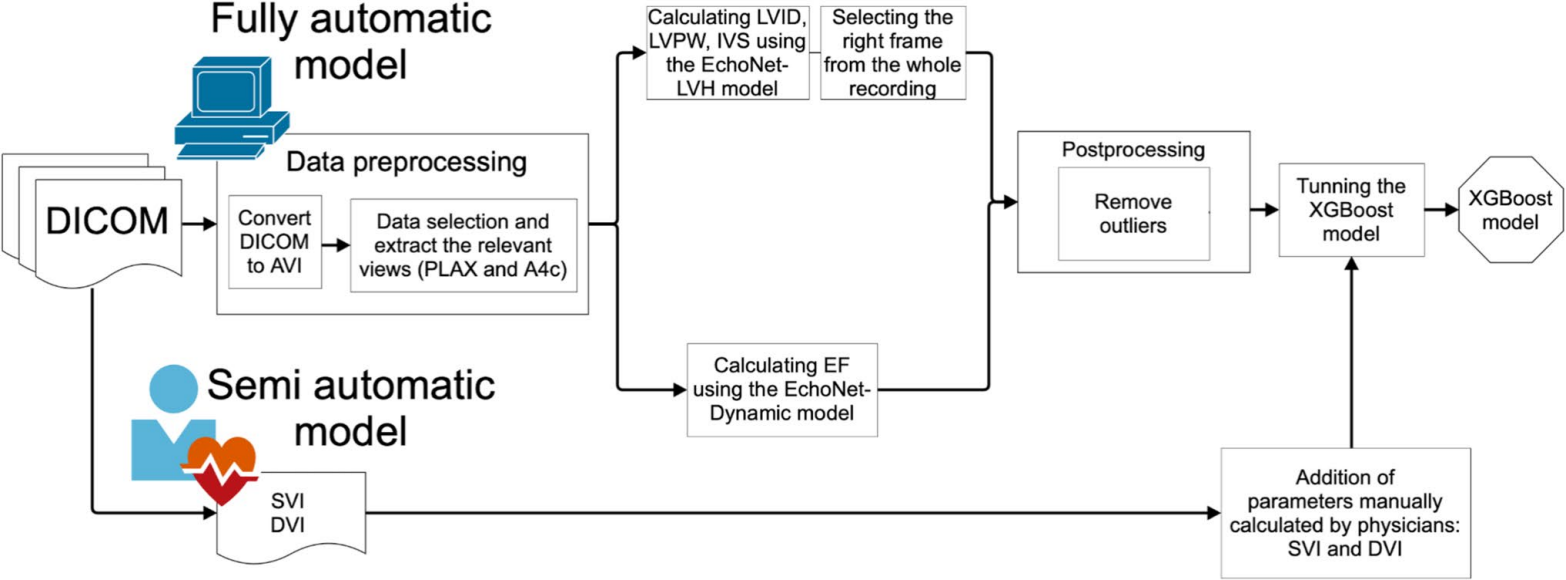
Diagnosing severe low-gradient aortic stenosis, as compared to moderate stenosis—a workflow

The image-derived parameters that were obtained automatically (Table 1) were used to train the AI model (extreme gradient boosting, XGBoost [17]) to differentiate between low-gradient severe and low-gradient moderate AS (Fig. 1). The hyperparameters were selected using the grid search technique. In a similar manner, semi-automatic models were created by additionally incorporating the manually calculated DVI and SVI parameters (Supplemental Fig. 1). The models were then subsequently compared with each other to assess the effectiveness of fully automatic, as compared to the semi-automatic approach.

### Model Validation

The ground truth used for model validation was the diagnostic information on AS subtype obtained with conventional medical means, i.e., with TTE, DSE, CT, or TEE. The stratified cross-validation was used to assess the quality of the model. The data, after being split into 10 equal folds, were used to train (9 folds) and to evaluate the performance of the developed model (one fold). The ratio of the severe to moderate AS cases was kept similar in each fold (i.e., stratified). This way, both conditions were similarly represented in the training and testing, which provides a more accurate estimate of the performance/error of the model. The in-build XGBoost’s gain measure was used to assess the feature importance of the parameters used in the models. The 95% confidence intervals (CIs) were calculated with a bootstrapping method. The differences in performance across models were assessed with the DeLong’s test [18].

### Statistical Analysis

Categorical variables were presented as frequencies (in percent), and continuous variables as median values (interquartile range) unless explicitly stated otherwise. To compare categorical variables, the Pearson χ^2^ statistic was used, and for the continuous variables, the Wilcoxon rank-sum or Kruskal–Wallis test was used.

The preprocessing steps, calculating the parameters—LVID, LVPW, and IVS with the EchoNet-LVH model and LVEF with EchoNet-Dynamic—as well as the cross-validation and training of XGBoost classifier were conducted on a computer configuration consisting of an Intel(R) Core(TM) i7 - 4770 K 3.50 GHz CPU, GeForce RTX 2070 8 GB GPU, 32 GB RAM. Analyses were performed using Python version 3.9.0.

## Results

### Preprocessing and Data Availability

As a result of the data quality analysis, 158 high-quality echocardiographic recordings were included in the study from the initial 174 exams, allowing for the evaluation of the feasibility of an automatic approach to diagnosing severe vs moderate AS. As for the semi-automatic approach, DVI and SVI parameters were available for 135 patients.

### Model Performance and Feature Importance

**Fig. 2:**
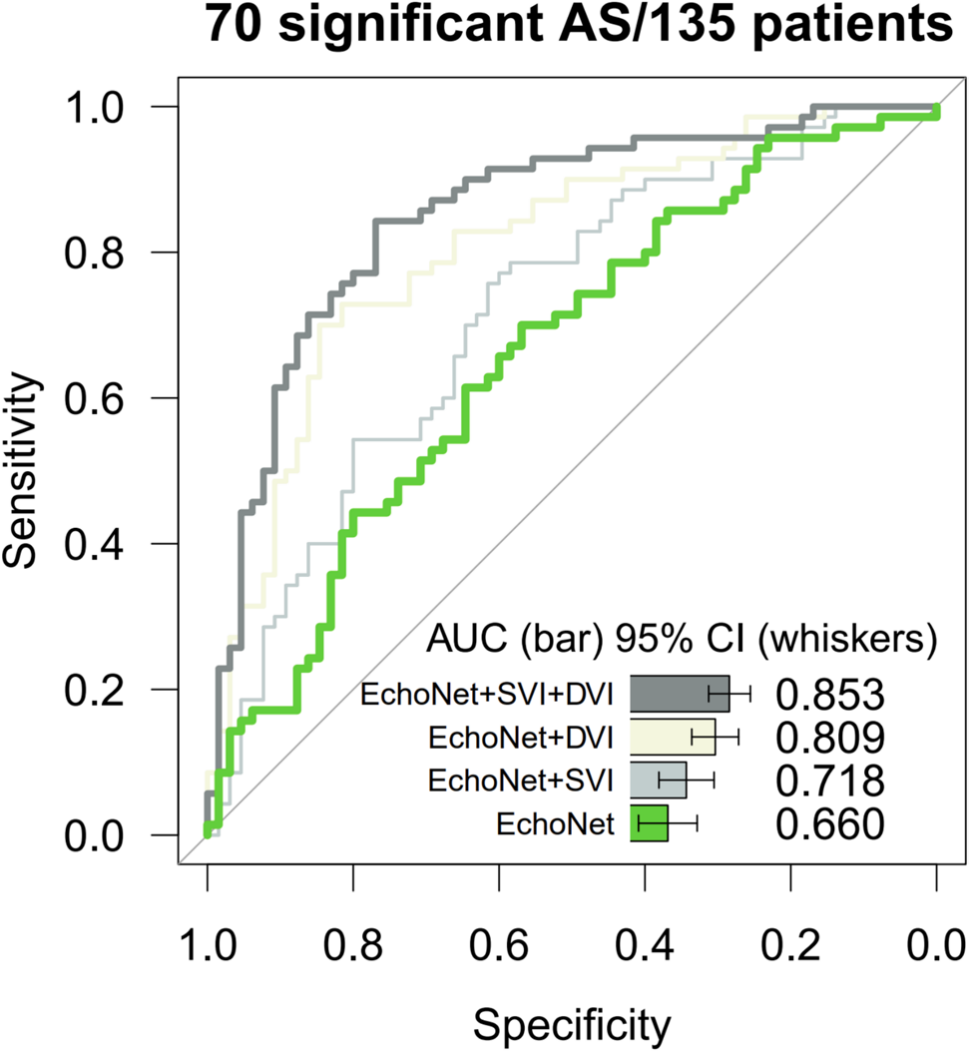
Comparison of models based on semi-automatic parameters and fully automatically derived parameters. SVI and DVI were manually obtained parameters; hence, the analysis is semi-automated. EchoNet was used to derive the parameters from the echocardiography images in a fully automatic manner

**Fig. 3:**
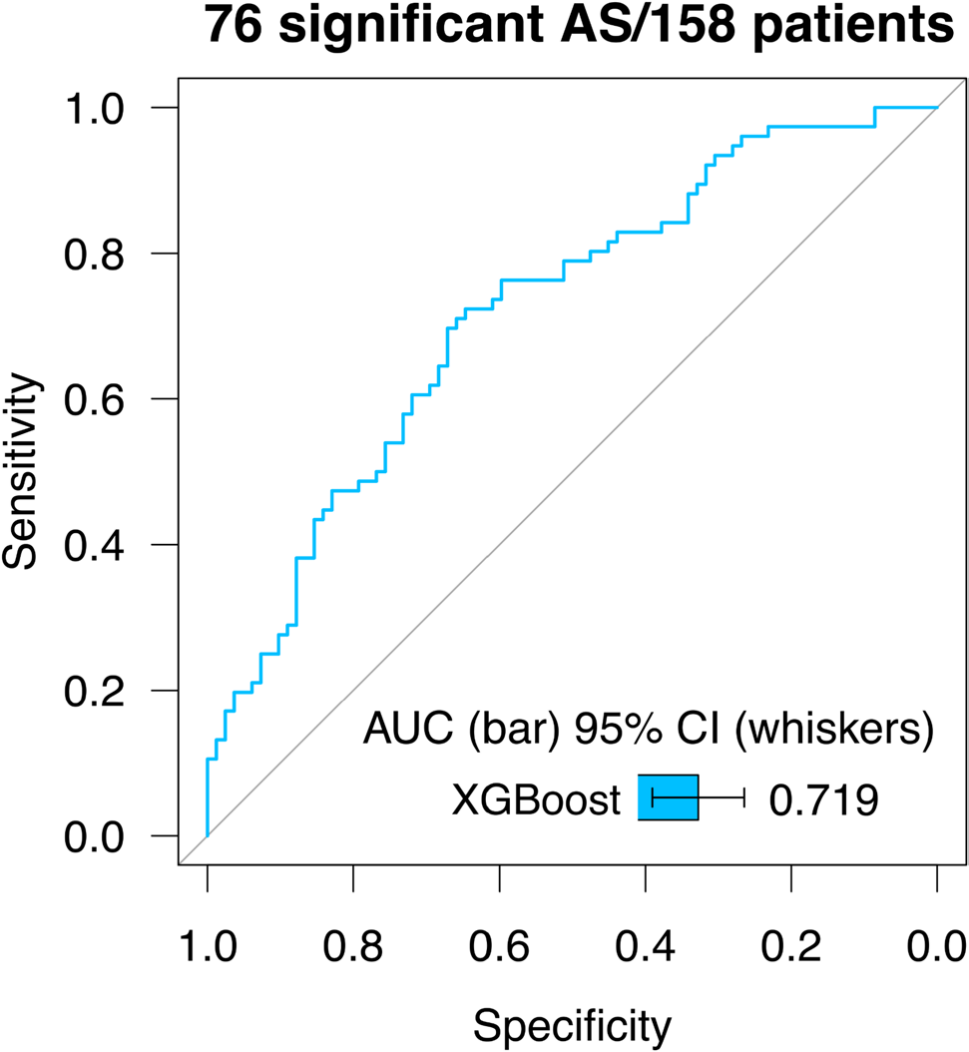
Results of the model trained on EchoNet parameters for 10-fold stratified cross-validation. This model was based on 4 automatically derived parameters

**Fig. 4:**
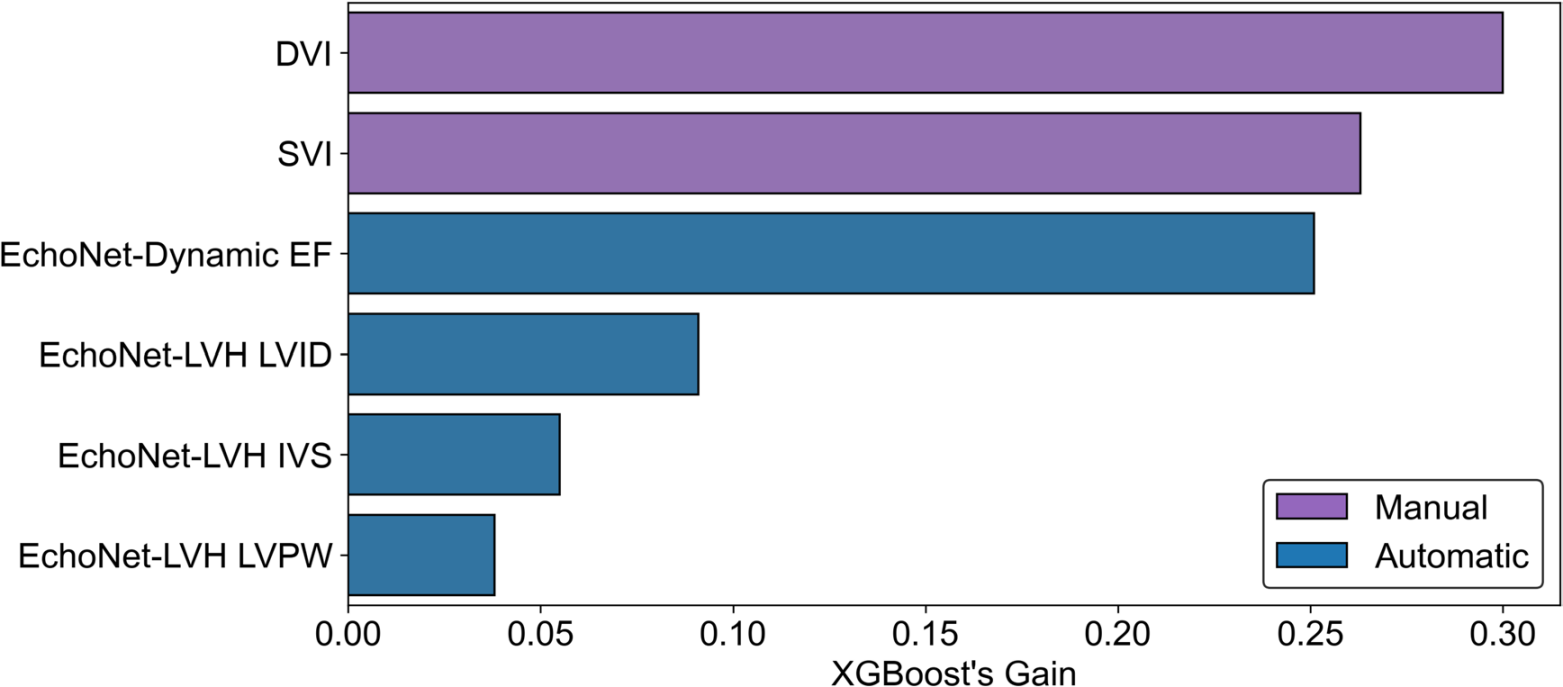
Feature importance of the parameters used in the models

The performance for the semi-automatic model, based on 6 parameters, i.e., LVID, LVPW, IVS, LVEF, SVI, and DVI, was 0.853 (95% CI: 0.788, 0.919), and on the same dataset of 135 patients, the performance for fully automatic approach (4 parameters used: LVID, LVPW, IVS, and LVEF) was 0.660 (95% CI: 0.568, 0.752) (DeLong’s test result, *p* value < 0.0005; Fig. 2). As for the semi-automatic approach, the results were: (1) EchoNet + SVI, AUC = 0.718 (95% CI: 0.632, 0.805), (2) EchoNet + DVI, AUC = 0.809 (95% CI 0.735, 0.882), and (3) EchoNet + SVI + DVI, AUC = 0.853 (95% CI: 0.788, 0.919). When data from all 158 patients, for which the 4 parameters that could be derived automatically were used, the performance increased to 0.719 (95% CI: 0.64, 0.798) (*p* = 0.34; Fig. 3). The performance of the model in atrial fibrillation and low LVEF subgroups of patients is presented in Supplemental Fig. 2. Of the features used in the model, the manually obtained DVI and SVI were the most important ones. They were followed by LVEF, LVID, IVS, and LVPW (Fig. 4).

## Discussion

In this work, we showed that with AI it is possible to differentiate between low-gradient severe and moderate AS, in an automatic manner, based only on imaging parameters. This way, multiple cumbersome medical procedures can potentially be replaced by an AI-based approach, in which only transthoracic echocardiography would be necessary to obtain image-derived parameters required by the model. Although our tests showed the semi-automatic method still yielded better results than the fully automatic one, by implementing even the semi-automatic procedure as a diagnostic decision support system, a significant burden of work could be alleviated from medical professionals, and inconveniences could be spared to the patients.

As aortic stenosis is one of the most common acquired heart diseases [2], and diagnosing it based on echocardiography readings has become a cornerstone of AI model development in cardiac sciences. To the best of our knowledge, although multiple groups have attempted AS diagnosis with AI methods [6–14, 19], our study is a first of its kind in which (1) imaging parameters, fully automatically derived from echocardiography recordings were used, i.e., no additional clinical parameters were considered; and (2) the AI model was used to distinguish between the most diagnostically challenging subtypes of AS, i.e., between the low-gradient severe and moderate AS [20].

Current medical practice is that determining the LG AS requires a comprehensive approach involving additional tests, such as dobutamine stress echocardiography (DSE), transesophageal echocardiography (TEE), or computed tomography (CT) which exposes the patient to additional risks associated directly with these procedures, such as radiation dose, and generates extra costs for the system. The solution developed and described in this article can help to avoid these cumbersome and costly procedures. The performance of our semi-automatic model (AUC = 0.853) shows that a reliable LG AS diagnostic means can be achieved in a faster and less expensive manner. The advantage of the semi-automatic over fully automatic approach (*p* < 0.0005) confirms the necessity to use manually derived flow parameters (SVI and DVI) for superior model performance. Nevertheless, calculating the DVI and SVI by a clinician based on a single echocardiographic examination (TTE) would still have an advantage over performing additional, more invasive medical procedures (e.g., DSE or TEE). Importantly, despite the inferiority of the fully automatic approach (AUC = 0.66; with no differences within AF and LVEF subgroups, Supplemental Fig. 2), it still can be potentially useful in clinical practice, e.g., in the case of patients for which obtaining reliable flow parameters is not possible.

Our fully automatic approach was based on the works by Duffy et al. and Ouyang et al., in which methods for automatic parameter derivation were developed [10–12]. Although severe AS diagnosis, as compared to non-AS individuals, was attempted before [10–12, 19], our work is the first to dwell into differentiating between more demanding low-grade stenosis cases. Interestingly, the Mouse-Echocardiography Neural Net (MENN) was recently developed to automate the analysis of echocardiograms in preclinical research [21]. This tool demonstrated an excellent correlation with manual analyses (Pearson’s *r* = 0.85–0.99) and reduced analysis time by over 92%, addressing inter-reader variability issues commonly faced in manual evaluations [21]. However, again, it was not used for low-gradient cases.

In the work by Sengupta et al., although the grade of severity of AS was distinguished in their study, this differentiation was not based on echocardiography data [22]. Additionally, Krishna et al. presented a fully automated AI model for assessing AS in echocardiography [23]. Although parameters obtained with AI were correlated with the measurements made manually by physicians (such as SVI), the model did not classify the subtypes of AS [23].

Manzo et al., in their review, described potential alternative parameters helpful in AS diagnosis, such as DVI or ejection dynamic parameters—acceleration time (AT) and the ratio of AT and ejection time (ET) [24]. We decided to use DVI in the semi-automatic approach as the use of this parameter is in line with current aortic valve guidelines [4]. The high performance of the semi-automatic model observed in our study shows that it is possible to reliably differentiate between moderate and severe low-gradient AS while using exclusively imaging parameters.

### Limitations

Unfortunately, our study had several limitations. The relatively small sample size of 174 studies limits the statistical power of the study and reduces the robustness of the model’s conclusions. However, given the high specificity of the selected cases, i.e., low-gradient severe AS vs moderate AS, this group can still be considered enough to get insights into model performance and an increase in its robustness, given the sample size, can follow (for studies on AS with similar sample sizes see [25, 26]). Additionally, there are possible differences between sexes in the AI model performance based on automatically derived parameters [27]. Finally, due to the sample used, the study may not account for regional or demographic variations in the patient general population, especially patients of races other than white.

## Conclusion

The model built on automatically derived left ventricle measurements, along with manually calculated SVI and DVI, has the potential to distinguish patients with severe AS in the group of the most challenging low-gradient aortic stenosis. Although the fully automatic approach performed worse than the semi-automatic one, the derivation of parameters such as SVI and DVI can be implemented in the future. The results presented in the current report hold a great promise in using AI models for differentiating between the most challenging subgroups of aortic stenosis, i.e., the severe low-gradient and moderate stenosis.

## Supplementary Information

Below is the link to the electronic supplementary material.

Supplemental Figure 1 Workflow of parameter derivation and model development for fully automatic and semi-automatic approaches. The graph presents for how many patients automatic parameters were available vs. manually-derived flow measurements. Also the model development and AUC obtaining process is presented (PNG 380 KB)

Supplemental Figure 2 Fully automatic performance in specific clinical subgroups. The model performance was compared between the subgroups of (A) patients with atrial fibrillation (AF), and without AF, as well as (B) with left ventricle ejection fraction (LVEF) lower than 50%, and equal or greater than 50% (PNG 249 KB)
