# Supplementary figures and images for: Diagnosing Severe Low-Gradient vs Moderate Aortic Stenosis with Artificial Intelligence Based on Echocardiography Images
Source: J Imaging Inform Med. 2025 Apr 21;39(1):926–32. doi: 10.1007/s10278-025-01497-4 (PMC12921054; doi:10.1007/s10278-025-01497-4)

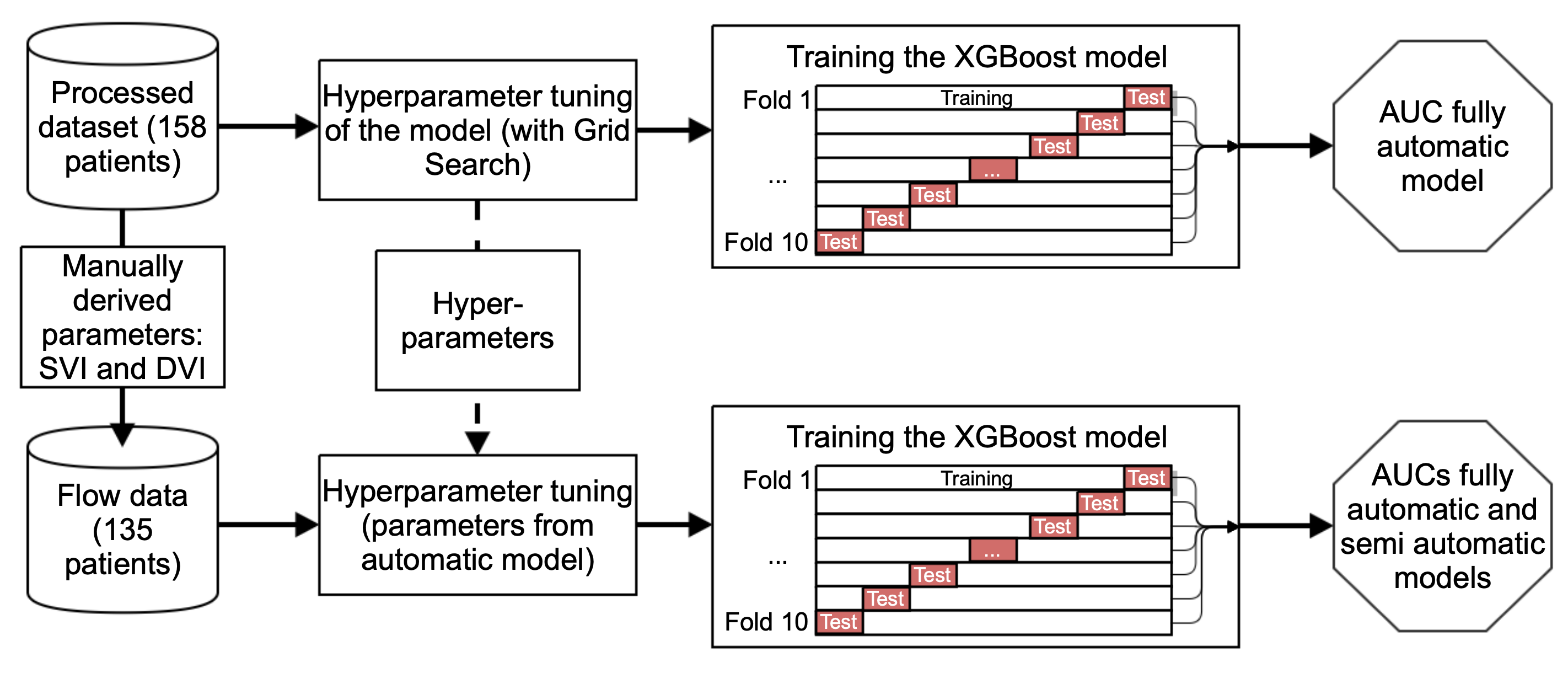

Supplement: Supplementary file 1 — Supplemental Figure 1 Workflow of parameter derivation and model development for fully automatic and semi-automatic approaches. The graph presents for how many patients automatic parameters were available vs. manually-derived flow measurements. Also the model development and AUC obtaining process is presented (PNG 380 KB) [file 10278_2025_1497_MOESM1_ESM.png]

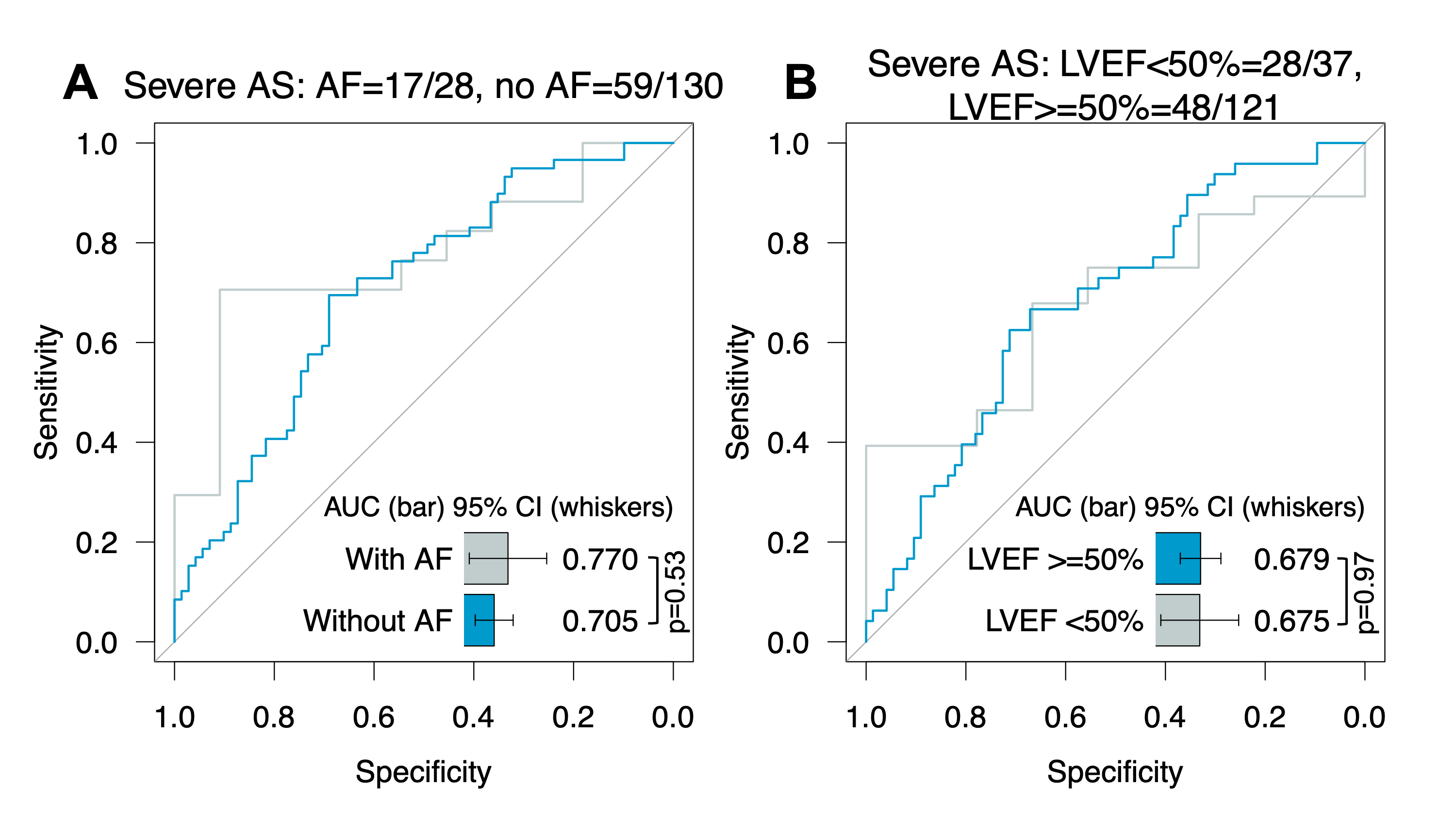

Supplement: Supplementary file 2 — Supplemental Figure 2 Fully automatic performance in specific clinical subgroups. The model performance was compared between the subgroups of (A) patients with atrial fibrillation (AF), and without AF, as well as (B) with left ventricle ejection fraction (LVEF) lower than 50%, and equal or greater than 50% (PNG 249 KB) [file 10278_2025_1497_MOESM2_ESM.png]
